# Glioma Stem Cells: Signaling, Microenvironment, and Therapy

**DOI:** 10.1155/2016/7849890

**Published:** 2016-01-06

**Authors:** Brandon D. Liebelt, Takashi Shingu, Xin Zhou, Jiangong Ren, Seul A. Shin, Jian Hu

**Affiliations:** ^1^Department of Neurosurgery, The University of Texas MD Anderson Cancer Center, Houston, TX 77030, USA; ^2^Department of Neurosurgery, Houston Methodist Neurological Institute, Houston, TX 77030, USA; ^3^Department of Cancer Biology, The University of Texas MD Anderson Cancer Center, Houston, TX 77030, USA

## Abstract

Glioblastoma remains the most common and devastating primary brain tumor despite maximal therapy with surgery, chemotherapy, and radiation. The glioma stem cell (GSC) subpopulation has been identified in glioblastoma and likely plays a key role in resistance of these tumors to conventional therapies as well as recurrent disease. GSCs are capable of self-renewal and differentiation; glioblastoma-derived GSCs are capable of *de novo* tumor formation when implanted in xenograft models. Further, GSCs possess unique surface markers, modulate characteristic signaling pathways to promote tumorigenesis, and play key roles in glioma vascular formation. These features, in addition to microenvironmental factors, present possible targets for specifically directing therapy against the GSC population within glioblastoma. In this review, the authors summarize the current knowledge of GSC biology and function and the role of GSCs in new vascular formation within glioblastoma and discuss potential therapeutic approaches to target GSCs.

## 1. Introduction

Glioblastoma is the most common and devastating primary brain tumor. The standard-of-care treatment involves maximal surgical resection followed by radiation and chemotherapy with temozolomide (TMZ) [1–4]. Despite treatment with rigorous surgical and medical therapy, patients only have a 15- to 19-month median overall survival rate because of near-universal tumor recurrence [4, 5]. Studies have emerged showing glioma stem cells (GSCs) to represent a subpopulation of cells within glioblastoma that are characterized by increased resistance to chemotherapy and radiotherapy, suggesting that GSCs are likely responsible for failure of treatment and high recurrence rates in glioblastoma [6]. Therefore, GSCs are considered a relevant target for glioblastoma therapy, and the elimination of GSCs is crucial in treating glioblastoma. The strategy to target GSCs therapeutically is mainly focused on the direct ablation of GSCs by targeting cell surface markers and specific pathways that are required for maintaining GSC stemness. However, it has been increasingly acknowledged that another way to specifically target GSCs is to alter the ability of GSCs to interact with their microenvironment, including their dependency on angiogenesis and their immune evasive properties. In this review, we summarize the current knowledge of GSC biology and function and discuss potential therapeutic approaches to target GSCs.

## 2. Cancer Stem Cell Biology

In their most basic definition, stem cells possess the ability to both self-renew and differentiate. Self-renewal is a critical function of stem cells, as they must persist throughout the entire lifespan of the organism. This quality of self-renewal is shared between both stem cells and cancer cells. Not surprisingly, there are several signaling pathways that have been identified, and likely numerous others yet to be identified, which are shared between these two cell types. Among these are the Notch, Sonic hedgehog (Shh), and Wnt signaling pathways [7]. These pathways are essential for preserving multipotency and self-renewal.

Cancer stem cells (CSCs) possess the same characteristics of normal stem cells with the added features of being oncogenic in their host and giving rise to a heterogeneous population of cells that comprise the tumor mass. These cells were first postulated as etiologic agents in hematopoietic cancers. Bonnet and Dick showed that the leukemia-initiating cell in acute myeloid leukemia possessed the characteristics of a leukemia stem cell. This showed that normal primitive cells, rather than committed cells, were capable of leukemic transformation [8]. Since then, these cells have also been identified in solid tumors, including prostate [9], colon [10], lung [11], ovarian [12], and brain [13] tumors. A pure CSC tumor model posits that a CSC forms the basis for tumorigenesis and continued propagation through self-renewal and differentiation into the various cellular types that comprise the tumor [14].

Neural stem cells (NSCs) can be found in several locations in the adult brain including the subventricular zone (SVZ) [15], dentate gyrus of the hippocampus [16], and the subcortical white matter [17]. The SVZ is presumed to be host to the majority of these cells and has been proposed as the site of origin of gliomas and other brain tumors [18], stemming from early experiments showing increased tumor formation after carcinogen injection into the SVZ compared to other sites in rats [19].

Further, cell cultures derived from human glioblastoma have been shown to have the ability to form neurospheres. Cells constituting the neurospheres were found to highly express both Nestin and CD133. These cells were also capable of* in vivo* tumor formation when injected into nude mice, whereas non-sphere-forming cells isolated from glioblastoma did not grow tumor [20]. Additional studies investigating gliomagenesis by exposure to chemicals (ethylnitrosourea) or viruses (avian sarcoma virus) in animals showed that tumor formation preferentially occurs in the SVZ, particularly with earlier exposure to the carcinogen, suggesting the importance of this site in the origin of gliomas [9, 21–23].

## 3. Implicated Signaling Pathways

**Figure 1:**
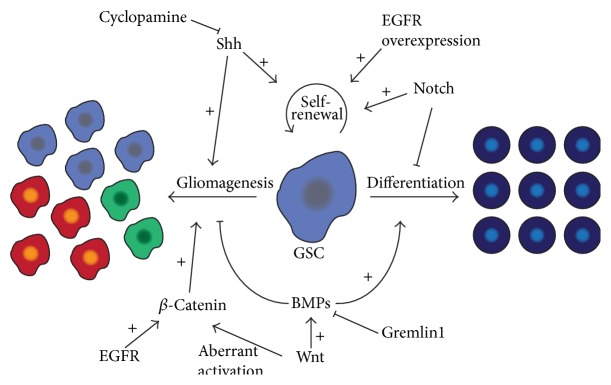
Summary of key molecular pathways regulating steps in glioma stem cell self-renewal, differentiation, and gliomagenesis.

Compared to NSCs, GSCs exhibit enhanced self-renewal capacity and compromised differentiation [24], summarized in Figure 1. GSCs upregulate a number of signaling pathways required for maintaining NSC stemness, which enables them to enhance their stemness and aberrant cell survival, consequently leading to tumorigenesis [25–27]. Therefore, further understanding the signaling pathways in normal neural development including Notch, bone morphogenic proteins (BMPs), NF-*κ*B, Wnt, epidermal growth factor (EGF), and Shh will give significant insight into the cellular features of GSCs and will aid in designing better treatment strategies for glioblastoma.

Notch signaling is important for mediating various cellular and developmental processes including the regulation of proliferation, differentiation, apoptosis, and cell lineage decisions in NSCs [28–30]. Recent studies have implicated Notch signaling to be highly active in GSCs to suppress differentiation and maintain stem-like properties. Downregulation of Notch and its ligands such as Delta-like-1 and Jagged-1 leads to decrease in oncogenic potential of GSCs, which indicates an important role of Notch signaling in GSC survival and proliferation [28, 31, 32].

BMPs regulate proliferation, differentiation, and apoptosis in NSCs. BMP signaling pathways are activated in different developmental processes depending on their interaction with various signaling molecules including Wnt/*β*-catenin, basic helix-loop-helix (bHLH), and hypoxia-inducible factor-1*α* (HIF-1*α*) [33–35]. Wnt signaling induces BMP expression, which predisposes NSCs toward an astroglial lineage [36]. Similarly, BMPs in GSCs are shown to play an important role in directing astroglial differentiation to inhibit the tumorigenic potential of GSCs [37]. Specifically, BMP-2 decreases GSC proliferation by directing astroglial differentiation and sensitizes GSCs to TMZ through destabilization of HIF-1*α* [34, 38].* In vivo* delivery of BMP-4 inhibits brain tumor growth with a resultant decrease in mortality [37]. A BMP antagonist, Gremlin1, inhibits differentiation of GSCs by its regulation of endogenous BMP levels to maintain GSC self-renewal and tumorigenic potential [39].

Wnt/*β*-catenin signaling is also important for regulating NSC expansion and promoting astroglial lineage differentiation in normal neural development [40, 41]. *β*-Catenin is a critical factor for proliferation and differentiation of GSCs [42, 43]. Aberrant activation of Wnt signaling in GSCs leads to tumor growth through nuclear localization of stabilized *β*-catenin [44, 45]. FoxM1/*β*-catenin interaction regulates the transcription of c-Myc and other Wnt target genes inducing glioma formation [46, 47]. In addition, Wnt/*β*-catenin signaling regulates the expression of PLAGL2 to suppress the differentiation of GSCs, maintaining their stemness [48].

The EGFR signaling pathway mediates proliferation, migration, differentiation, and survival in NSCs [49–53]. Levels of EGFR expression vary with specific stages of development, which suggests a requirement for precise modulation of EGFR expression by balancing extrinsic signals such as BMP and FGF during normal neuronal development [54]. EGFR activation promotes GSC proliferation and tumorigenesis by transactivation of *β*-catenin [55]. Furthermore, overexpression of EGFR increases the self-renewal capacity of GSCs resulting in induction of their tumorigenic potential [56–59].

Sonic hedgehog (Shh) signaling is pivotal in ventral patterning, proliferation, differentiation, and survival of NSCs [60–62]. In the adult brain, persistent Shh pathway signaling in the SVZ is critical for the regional specification and maintenance of NSCs [63, 64]. Recent studies demonstrate that the Shh pathway is highly active in GSCs to maintain self-renewal and induce tumorigenesis by regulating stemness genes. Suppression of Shh signaling reduces self-renewal and* in vivo* tumorigenicity, which indicates the dependency of GSCs on Shh signaling for their survival [65, 66].

## 4. Microenvironment of GSCs: Vasculature in Glioma

Neovascularization in malignant glioma is well documented, being characterized as hypervascular tumors associated with aberrant vascular morphology [67–69]. Normal vessels are formed by the mechanisms of vasculogenesis and angiogenesis [67, 70]. Vasculogenesis is* in situ* vascular formation through differentiation of mesodermally derived endothelial progenitor cells, angioblasts, which occurs during organogenesis and fetal development. Formation of a primitive vascular network by vasculogenesis is followed by angiogenesis, which contributes to expansion and remodeling of the existing vasculature by two different mechanisms: branching by vessel sprouting (sprouting angiogenesis) and splitting of vessel lumens by interstitial tissue (intussusception). Other than vasculogenesis and angiogenesis, malignant gliomas exhibit two additional types of neovascularization: vascular co-option and vasculogenic mimicry [67–69]. Recent studies implicate roles of GSCs in multiple modes of glioma neovascularization.

### 4.1. Vascular Co-Option

Glioma cells infiltrate around normal brain vessels forming perivascular cuffs, incorporating the existing vessels into the tumor in a process called vascular co-option [71]. Although the specific role of GSCs in vascular co-option has not been established, vascular co-option is followed by apoptosis of endothelial cells and regression of vessels. This results in hypoxia that in turn induces angiogenesis in which GSCs play critical roles [72].

### 4.2. Angiogenesis

Angiogenesis is the process of new vessel development from preexisting vasculature, with VEGF playing a critical role in this process [67]. Several studies suggest critical roles of GSCs in glioma angiogenesis. Conditioned medium from CD133+ GSCs contains 10–20-fold higher levels of VEGF than that from CD133− cells and promotes human microvascular endothelial cell migration and tube formation [73]. Hypoxia stimulates production of VEGF and stromal cell-derived factor 1 (SDF-1), also known as CXCL12, in GSCs [74, 75]. VEGF induces migration and proliferation of endothelial cells, while SDF-1 causes migration of endothelial cells [76]. GSCs also secrete hepatoma-derived growth factor that promotes endothelial cell migration* in vitro* and angiogenesis* in vivo* [77].

### 4.3. Vasculogenesis

Vasculogenesis was originally described as* de novo* vascular formation by angioblasts derived from mesoderm during organogenesis and fetal development, and it had been believed that postnatal vasculature could be formed only by angiogenesis even in pathological conditions [69, 70, 78]. This theory was challenged by findings of tumor vasculogenesis by Asahara et al., who reported formation of tumor vessels by circulating endothelial cell progenitors [79]. Several subsequent studies indicated that other types of cells including tumor-associated macrophages (TAMs)/Tie-2 expressing monocytes and GSCs also differentiate into endothelial cells in the tumor [80–84]. Although there is controversy regarding what is referred to by “vasculogenesis,” we will use vasculogenesis to describe any* de novo* neovascularization. VEGF and SDF-1*α* were overexpressed by GSC-rich C6 rat glioma cells in culture [76]. Inhibition of VEGF or SDF-1*α* suppressed endothelial cell proliferation, tubule formation, and endothelial progenitor cell mobilization and decreased vascularization, suggesting an important role of GSCs in not only angiogenesis but also vasculogenesis. Ricci-Vitiani et al. reported that some CD31+ endothelial cells in human glioblastoma specimens carried the same chromosomal aberrations as tumor cells. CD133+ GSCs cultured in endothelial conditions generated CD31+ and Tie-2+ endothelial cells, and vessels in tumors formed by GSCs in immunocompromised mice were mainly composed of human CD31+ endothelial cells [83]. Wang et al. reported that glioblastoma-derived CD133+ cells included a CD144+ (vascular E-cadherin) population. These CD133/CD144 double positive cells showed an increase in expression of CD31, CD105, CD34, and VEGFR-2 and decrease in CD144 expression under endothelial culture conditions and were capable of forming a tubular network in Matrigel [85]. Finally, Soda et al. demonstrated that glioma tumor-initiating cells produced endothelial cells expressing CD31, CD34, CD144, and von Willebrand factor in a genetically engineered mouse brain tumor model [86]. Although the selection and interpretation of marker proteins for endothelial progenitor cells and endothelial cells are not identical, their findings suggest transdifferentiation of GSCs into endothelial cells. However, the biological and clinical significance of glioma vasculogenesis is still in debate. It was shown that only 10% of the vessels were identified to contain cells expressing neoplastic markers, and when identified these cells comprised less than 10% of the vascular cellularity in the cross section of human glioblastoma [87]. A study using chimeric mice with GFP-tagged bone marrow cells also showed that less than 1% of bone marrow-derived cells were incorporated into the vascular endothelial layer in experimental gliomas [88]. Taken together, incorporation of bone marrow-derived or GSC-derived cells into vascular endothelium may be a rare event or widely vary among tumors [67]. However, it is possible that vasculogenesis could play a critical role in glioma resistance to antiangiogenic therapy and early revascularization events in recurrent glioma [68].

Recent studies suggest that pericytes play a critical role not only in physiological processes such as wound healing but also in tumor growth and progression [89]. Furthermore, it was reported that GSCs give rise to pericytes [90, 91]. Although the role of GSC-derived pericytes in glioma neovascularization remains to be clarified, the finding that targeting GSC-derived pericytes suppressed neovasculature formation and tumor growth suggests an important function of these cells in glioma vascularization and progression.

### 4.4. Vasculogenic Mimicry

Vasculogenic mimicry is a fluid-conducting, matrix-embedded meshwork that is formed not by endothelial cells but by tumor cells [92]. This finding has been observed in human malignant melanoma specimens [93] and documented in malignant astrocytoma [94, 95].

A study using human GSCs and GFP transgenic nude mice showed formation of patterned, tubular networks of vascular channels formed by human GSC-derived cells in xenograft tumors [96]. CD133+ GSCs established from human glioblastoma have successfully formed a vasculogenic network in a 3D Matrigel tube formation assay [97]. Similarly, Chen et al. demonstrated* in vitro* vascular formation by CD133+ GSCs with formation of vasculature lined by nonendothelial cells [98]. Knockdown of VEGFR-2 in GSCs inhibited formation of tubules, xenograft tumors, and vasculogenic mimicry [99]. In hypoxic conditions (1% O_2_), CDH5 (CD144) was upregulated by HIF-1*α* and HIF-2*α* in GSCs and contributed to vasculogenic mimicry [100].

Although the extent of contribution of these mechanisms to glioma neovascularization seems to vary among tumors, GSCs are thought to contribute to at least three of the above four mechanisms. Additionally, GSCs can also transdifferentiate into pericytes that support the tumor vasculature.

## 5. GSC-Dependent Therapeutic Resistance of Glioblastoma

A major challenge for glioblastoma treatment is radioresistance and chemoresistance of the recurrent tumor, possibly due to an increased population of GSCs after initial treatment [101–103]. Increasing evidence shows that GSCs contribute to recurrence and therapy resistance through multiple mechanisms, such as alteration of DNA damage responsive machineries, hypoxic microenvironment, Notch signaling pathway, and multidrug-resistance mechanisms [6, 104–106]. On the other hand, these findings also offer a novel opportunity for therapeutic intervention on GSCs in glioblastoma patients.

Ionizing radiation represents an effective therapeutic option for glioblastoma by inducing DNA damage. Thus, DNA damage responses play crucial roles in cellular radiosensitivity and radioresistance [107]. To date, the underlying mechanism of radioresistance in glioblastoma remains elusive. Bao et al. observed that CD133+ GSCs were enriched after radiation, while CD133− cells were more sensitive to ionizing radiation [6]. Mechanistically, they found that CD133+ cells preferentially activated the DNA damage checkpoint in response to radiation and thus repaired DNA damage more efficiently [6]. Moreover, inhibitors of Chk1 and Chk2 checkpoint kinases could restore the radiosensitivity of CD133+ GSCs [6]. However, a study by McCord et al. showed conflicting results. They found that all six lines of CD133+ glioblastoma stem-like cells were more sensitive to radiation than the established glioma cell lines [108]. They also found that the CD133+ glioblastoma stem-like cells showed a significantly reduced DNA repair capacity [108]. One possible explanation for the contradictory observations between these two studies may be that established glioma cell lines (U87 and SF-126), but not paired CD133− cells, were used as controls in McCord's study. TMZ, a commonly used alkylating agent, undergoes pH-dependent hydrolysis to its reactive compound 5-3-(methyl)-1-(triazen-1-yl) imidazole-4-carboxamide (MTIC) in cells, which causes DNA damage by methylating the O6-position or N7-position of guanine [109]. The methyl adducts lead to a continuous cycle of DNA base mismatch repair (MMR), resulting in double strand breaks and eventual apoptosis. Increasing evidence demonstrates that the O6-methylguanine methyltransferase (MGMT), whose function is repairing the mutagenic DNA lesion O6-methylguanine back to guanine, is expressed in 80% of glioblastoma patients [110]. MGMT plays an important role in resistance to TMZ, and glioblastoma patients carrying a methylated MGMT promoter exhibit improved progression-free and overall survival after treatment with alkylating agents [110, 111]. Other signaling pathways such as (JNK) or microenvironment conditions (hypoxia) can also contribute to chemoresistance of glioblastoma by upregulating the expression of MGMT [112, 113]. Therefore, a better understanding of MGMT and DNA repair responses will help to delineate the detailed mechanisms of radioresistance and chemoresistance of GSCs.

A number of signaling pathways, including Notch, Shh, and receptor tyrosine kinase (RTK) signaling, have also been implicated in therapy resistance of glioblastoma. For example, *γ*-secretase inhibitors (GSIs) that inhibit the Notch pathway sensitize GSCs but not nonstem glioma cells to radiation [114]. In addition, overexpression of the constitutively active form of Notch1 or Notch2 rendered GSCs much more resistant to radiation [114]. A previous study has shown that Shh-GLI signaling regulates the stemness of CD133+ GSCs, and cyclopamine, an inhibitor of Shh, displays synergistic effects with TMZ on GSC proliferation and apoptosis [66]. Another study confirmed that combination of either Notch inhibitor or hedgehog inhibitor, with temozolomide, enhanced the cytotoxicity on GSCs, and a significant effect was observed when the GSCs were treated with all three drugs simultaneously [115]. Abnormal activation of RTKs has been found in glioblastoma, such as PDGFR*α* in the proneural subtype and EGFR in the mesenchymal subtype [116]. Multiple RTKs and their involved signaling pathways are coactivated, leading to limited efficacy to therapy against single RTKs [117].

Environmental factors, like local cytokines and hypoxia, are crucial aspects of the microenvironment in glioblastoma and are generally correlated with worse prognosis. Among these extrinsic environmental factors, hypoxia has been attributed to play an important role in chemoresistance of GSCs. A recent study demonstrated that increased numbers of GSCs are localized in the core of the tumor mass along the intratumoral hypoxic gradient [118]. The hypoxic conditions help to maintain the undifferentiated state of GSCs through hypoxia-inducible factor-2*α* (HIF-2*α*) and multiple HIF-2*α*-induced genes [119, 120]. Importantly, markers related to chemoresistance (TIMP-1 and MGMT) were also highly expressed in the GSCs of the inner tumor [118, 121]. Another study found that elevation of MGMT expression via HIF-1*α* in GSCs contributes to its chemoresistance [113]. The observation that a hypoxia-driven undifferentiated state contributes to the chemoresistance of glioblastoma compels further effort to define the mechanisms of chemoresistance in GSCs and look for novel therapeutic approaches to target GSCs under the hypoxia niche effectively.

Recurrent glioblastoma exhibits resistance to multiple therapeutic drugs, leading to a hypothesis that GSCs are naturally resistant to chemotherapy. One potential explanation is that GSCs can reduce drug uptake or expel cytotoxic drugs by increasing the expression of ATP-binding cassette (ABC) transporter [122]. A recent study suggested that the PTEN/PI3K/Akt pathway could regulate ABCG2 activity in glioma cancer stem-like cells [106]. The authors also showed that loss of PTEN or treatment with TMZ increased the GSC population [106]. Another possibility for chemoresistance of GSCs is that GSCs exhibit abnormalities of cell death pathways, such as overexpression of antiapoptotic proteins or downregulation of proapoptotic factors [123]. Further efforts need to be devoted to understanding the molecular mechanisms of chemoresistance in GSCs and developing novel and effective therapeutic approaches against GSCs.

## 6. GSC-Targeted Therapies

### 6.1. Therapeutic Targeting of GSCs by Surface Markers

CD133 is one of the best characterized cell surface makers in GSCs and NSCs. CD133+ cells in glioblastoma display cancer stem cell-like properties and CD133 is known to be highly expressed in GSCs [124]. Furthermore, patients with high levels of CD133 show poor clinical outcomes [125]. Thus, therapies against CD133 might represent a promising strategy for glioblastoma treatment. Brescia et al. reported that disruption of CD133 expression by short hairpin RNA in human glioblastoma neurospheres impaired the self-renewal and tumorigenic capacity of neurosphere cells [124]. Further, treatment with carbon nanotubes conjugated to an anti-CD133 monoclonal antibody followed by irradiation with near-infrared laser light can selectively target CD133+ glioblastoma cells, and the photothermolysis caused by the nanotubes can kill targeted cells [126]. Recently, Emlet et al. reported that EGFRvIII is highly coexpressed with CD133 and EGFRvIII+/CD133+ defines the population of GSCs with the highest degree of self-renewal and tumor-initiating ability. Elimination of the EGFRvIII+/CD133+ population using a bispecific antibody could reduce tumorigenicity of implanted tumor cells, and the combined effect is better than any reagent directed against a single epitope [127].

L1 cell adhesion molecule (L1CAM, CD171) is a regulator of cell survival and is preferentially expressed on CD133+ GSCs [128, 129]. Bao et al. reported that shRNA-mediated knockdown of L1CAM decreased the sphere-forming ability and induced apoptosis of CD133+, but not CD133−, glioma cells* in vitro*. L1CAM knockdown in CD133+ glioma cells prior to xenotransplantation into immunodeficient mice markedly inhibited* in vivo* tumorigenesis and prolonged survival of the xenograft recipients. Mechanistically, L1CAM knockdown decreased the expression of bHLH transcription factor and upregulated p21WAF1/CIP1 tumor suppresser in CD133+ glioma cells. Furthermore, intracranial administration of lentiviral shRNAs against L1CAM in glioma xenografts also substantially suppressed tumor growth and prolonged survival of the tumor-bearing mice [130]. Together, these data suggest that L1CAM is required for maintaining the growth and survival of CD133+ glioma cells both* in vivo* and* in vitro*, and L1CAM may represent a GSC specific therapeutic target for improving the treatment of glioblastoma and possibly other brain tumors.

However, despite these efforts relying on CD133 staining, recent studies indicate that CD133+ tumor cells cannot simply be considered GSCs because not all GSCs express CD133, and subgroups of glioblastoma driven by CD133**−** GSCs have recently been identified [131, 132]. Therefore, further work is needed to confirm the role of CD133 in GSCs and identify more optimal markers for GSCs. This will not only enhance our knowledge of GSCs but also give us additional understanding of effective ways to target these cells.

### 6.2. Therapeutic Targeting of GSCs by Signaling Pathway

Signaling pathways, such as Notch, Shh, VEGF, STAT3, and BMP, are important for regulating GSC self-renewal and differentiation. Therefore, targeting these signaling pathways and their receptors in GSCs holds promise for glioblastoma therapy. Notch signaling is known to promote the survival and proliferation of NSCs and to inhibit differentiation [133]. Fan et al. reported that inhibiting Notch activation by *γ*-secretase inhibitors (GSIs) resulted in diminished proliferation, increased neuronal differentiation, reduced CD133+ cell fraction* in vitro*, and decreased tumorigenicity* in vivo* [134]. Shh pathway is also highly expressed in both glioblastoma and cell lines, and Shh ligand is expressed in glioblastoma-derived neurospheres. Treating glioblastoma-derived neurospheres with Shh inhibitor cyclopamine diminished new neurosphere formation, and viable glioblastoma cells injected intracranially following Shh blockade were no longer able to form tumors in athymic mice [65]. STAT3 pathway is required for GSC maintenance partially through upregulating TLR9 expression [135, 136]. Herrmann et al. reported that stimulation of TLR9 with a CpG ligand (CpG ODN) activated STAT3 pathway signaling and promoted GSC growth, whereas silencing TLR9 expression abrogated GSC development [137].

Other than targeting the stemness signaling of GSCs, inducing GSCs differentiation is another approach that has been tested to target GSCs. Piccirillo et al. reported that activating BMP signaling could differentiate GSCs in experimental models of human glioblastoma [37]. Administration of BMP4 to human glioblastoma-bearing mice induced CD133+ GSC differentiation and markedly attenuated CD133+ GSC sphere-forming frequency [37]. In addition, implantation of BMP4-treated glioblastoma xenografts to murine recipients resulted in smaller tumor lesions and substantially prolonged host survival compared with untreated controls [37]. Therefore, BMP4 may act as a key inhibitory regulator of gliomagenesis and be used in combined stem cell-based therapy as a noncytotoxic therapeutic agent.

### 6.3. Therapeutic Targeting of the Tumor Microenvironment

Since the tumor microenvironment is essential for maintaining GSC stemness, targeting the microenvironment is a promising approach for treating glioblastoma. The glioblastoma microenvironment mainly is composed of microvasculature and TAMs. VEGF level has been recognized to correlate with microvasculature formation and tumor growth [138]. Recognition of the VEGF pathway as a key regulator of angiogenesis has led to the development of several VEGF-targeted agents such as bevacizumab. Calabrese et al. have treated mice bearing U87 glioma cell xenografts with bevacizumab and observed a reduction in the number of CD133+/Nestin+ tumor-initiating cells, decreased microvasculature density, and decreased tumor growth [139]. Numerous studies have been reported showing that TAMs are enriched in glioblastoma and are very important components of the tumor microenvironment [140–144]. M2 TAMs could facilitate glioblastoma tumor growth by promoting neovascularization and play a tumor-supportive role in glioblastoma progression [145]. Recently, Zhou et al. reported that GSCs secrete periostin (POSTN) to recruit TAMs to support glioblastoma progression [146]. Silencing POSTN in GSCs markedly reduced TAM density, inhibited tumor growth, and increased survival of mice bearing GSC-derived xenografts. These studies indicate that targeting the interaction between GSCs and their microenvironment might represent an alternate approach in glioblastoma therapy.

## 7. Conclusion

In summary, glioblastoma remains a particularly challenging disease as little progress has been made towards improving patient outcomes and survival. A better understanding of the origins of this cancer and the molecular biology driving gliomagenesis is needed to tailor therapy towards addressing the root cause of this disease. Directly targeting glioma stem cells and their microenvironment presents a promising opportunity to eliminate the likely source of gliomas and the nidus of their recurrence.
